# Relationship between Semi-Quantitative Parameters of Thallium-201 Myocardial Perfusion Imaging and Coronary Artery Disease

**DOI:** 10.3390/diagnostics10100772

**Published:** 2020-09-30

**Authors:** Chin-Chuan Chang, Ming-Hui Yang, Chih-Ting Liu, Hsiu-Lan Chu, Chia-Yang Lin, Wei-Jheng Yen, Chao-Yu Chung, Sheng-Yow Ho, Yu-Chang Tyan

**Affiliations:** 1 Department of Nuclear Medicine, Kaohsiung Medical University Hospital, Kaohsiung 80756, Taiwan; chinuan@gmail.com (C.-C.C.); sindysmile@yahoo.com.tw (C.-T.L.); 740144@ms.kmuh.org.tw (H.-L.C.); 950195@ms.kmuh.org.tw (C.-Y.L.); yanweijheng@gmail.com (W.-J.Y.); 2 Department of Electrical Engineering, I-Shou University, Kaohsiung 84001, Taiwan; 3 School of Medicine, College of Medicine, Kaohsiung Medical University, Kaohsiung 80708, Taiwan; 4 Neuroscience Research Center, Kaohsiung Medical University, Kaohsiung 80708, Taiwan; 5 Department of Medical Education and Research, Kaohsiung Veterans General Hospital, Kaohsiung 81362, Taiwan; w3e3@hotmail.com; 6 Department of Chemistry, National Sun Yat-sen University, Kaohsiung 80424, Taiwan; cychung@mail.nsysu.edu.tw; 7 Department of Radiation Oncology, Chi Mei Medical Center, Tainan 71004, Taiwan; shengho@seed.net.tw; 8 Graduate Institute of Medical Science, Chang Jung Christian University, Tainan 71101, Taiwan; 9 Department of Medical Imaging and Radiological Sciences, Kaohsiung Medical University, Kaohsiung 80708, Taiwan; 10 Graduate Institute of Medicine, College of Medicine, Kaohsiung Medical University, Kaohsiung 80708, Taiwan; 11 Institute of Medical Science and Technology, National Sun Yat-Sen University, Kaohsiung 80424, Taiwan; 12 Department of Medical Research, Kaohsiung Medical University Hospital, Kaohsiung 80756, Taiwan; 13 Center for Cancer Research, Kaohsiung Medical University, Kaohsiung 80708, Taiwan; 14 Research Center for Environmental Medicine, Kaohsiung Medical University, Kaohsiung 80708, Taiwan; 15 Graduate Institute of Animal Vaccine Technology, National Pingtung University of Science and Technology, Pingtung 91201, Taiwan

**Keywords:** stress perfusion deficit, semi-quantitative parameters, thallium-201, myocardial perfusion imaging, coronary artery disease

## Abstract

This study aimed to investigate the diagnostic performance of semi-quantitative parameters of thallium-201 myocardial perfusion imaging (MPI) for coronary artery disease (CAD). From January to December 2017, patients were enrolled who had undergone Tl-201 MPI and received cardiac catheterization for coronary artery disease within three months of MPI. Receiver operating characteristics (ROC) analysis was used to determine the optimal cutoff values of semi-quantitative parameters. A comparison of the sensitivity and specificity of these parameters based on different subgroupings was further performed. A total of 130 patients were enrolled for further analysis. Among the collected parameters, the stress total perfusion deficit (sTPD) had the highest value of the area under curve (0.813) under the optimal cutoff value of 3.5%, with a sensitivity and specificity of 73.5% and 74.5%, respectively (*p* = 0.0000), for the diagnosis of CAD. With further subgrouping analysis based on history of diabetes or dyslipidemia, the sensitivity and specificity showed similar results. Based on the currently collected data and image acquisition conditions, the sTPD parameter has a clinical role for the diagnosis of CAD with a cutoff value of 3.5%.

## 1. Introduction

Coronary artery disease (CAD) is a leading cause of death among adults in the developed world [1]. The risk factors of CAD include high blood pressure, diabetes, cigarette smoking, a high level of low-density lipoprotein (LDL) cholesterol and low levels of high-density lipoprotein (HDL) cholesterol [2,3,4]. For the diagnosis of CAD, clinical history, blood tests and electrocardiograms are the preliminary examination methods, and cardiac catheterization is the gold standard of diagnostic examination to determine coronary artery disease. However, cardiac catheterization is an invasive procedure, which may confer certain mortality and morbidity rates in clinical practice [5]. On the contrary, radionuclide myocardial perfusion imaging (MPI), the most commonly used non-invasive cardiologic examination in Nuclear Medicine [6,7], plays an important role in detecting the status of coronary artery perfusion in the early clinical setting and helps to diagnose CAD.

In the past 40 years, MPI has been widely used in the detection and evaluation of coronary artery disease. The inspection methods have changed; for example, they depend on different inducers of the stress phase (dipyridamole, adenosine or exercise) [8], different radiopharmaceuticals (thallium-201 or technetium-99m methoxyisobutylisonitrile, MIBI) [9] and different workflows (single-day or two-day examination) [10], etc. As to the qualitative and quantitative interpretation of MPI, some institutes use the subjective visual tomographic analysis to determine the degree and extent of reversible or fixed perfusion defects for radiopharmaceuticals. The subjective visual interpretation is qualitative and may lead to uncertain diagnostic results due to interobserver bias. A variety of highly specific and reproducible semi-quantitative parameters generated by commercially available software [11] have made up for the shortcomings and enhanced the sensitivity, specificity and accuracy of detection [12,13,14].

Many different semi-quantitative parameters are currently being developed, including a 17-segment, five-point scoring system [15], the left ventricular ejection fraction (LVEF) after gated acquisition [16], the transient ischemic dilation (TID) of the left ventricle [17], the lung-to-heart ratio (LHR) [18], and the total perfusion deficit (TPD) [19], etc. Each parameter has its own cutoff value, and the cutoff is different according to the different inspection methods, processes or other related factors. To date, most of the relevant studies have been to compare the correlation between one or two semi-quantitative parameters and CAD, and Tl-201 has been rarely used as the radiopharmaceutical [20,21,22,23].

Therefore, the goal of this study was to compare the semi-quantitative parameters obtained by Tl-201 myocardial perfusion imaging with the results of cardiac catheterization. We tried to find the best cutoff values for the semi-quantitative parameters so that the imaging results could provide more diagnostic accuracy.

## 2. Materials and Methods

### 2.1. Patient Population

The study was conducted retrospectively to analyze the medical records of patients who received single-day, stress and resting Tl-201 myocardial perfusion imaging in the Nuclear Medicine Department in Kaohsiung Medical University Hospital. The inclusion criteria were as follows: patients who (a) had undergone a single-day, stress and testing Tl-201 myocardial perfusion study, (b) were aged over 18 years, and (c) had had cardiac catheterization confirmation for coronary artery disease within 3 months of myocardial perfusion imaging. Patient consent was waived because all of the clinical data were collected via the review of medical charts retrospectively. However, written permission from patients upon the clinical visit and examination were acquired. The study design was approved by the Institutional Review Board in our hospital (KMUHIRB-E(I)-20180299, Date: 9 November 2018). Clinical data were collected from January to December 2017.

### 2.2. Myocardial Perfusion Imaging

Before the myocardial perfusion imaging, patient baseline blood pressure was recorded. Dipyridamole with a dosage of 0.56 mg per kilogram body weight was used to induce the vasodilatation of coronary arteries. With electrocardiogram (ECG) monitoring, a slow intravenous injection of dipyridamole for 4 min was performed. Four minutes later, 3 mCi of the radioisotope Tl-201 was then injected. Single photon emission computed tomography (SPECT/CT) imaging was performed with an L-shaped, double-headed gamma-camera (BrightView, SPECT Gamma camera, Philips) equipped with a low-energy, high-resolution collimator. The collection conditions were 180-degree arc photography with step capture from the patient′s right anterior oblique (RAO) position to the left posterior oblique (LPO) position (RAO 45 degrees to LPO 45 degrees). The matrix size of each image was 64 × 64, and one set was taken for 60 s to collect 32 plane images. Three to four hours after the stress imaging acquisition, the patients underwent resting imaging with the same acquisition conditions as for the stress imaging.

### 2.3. Semi-Quantitative Parameters

**Figure 1:**
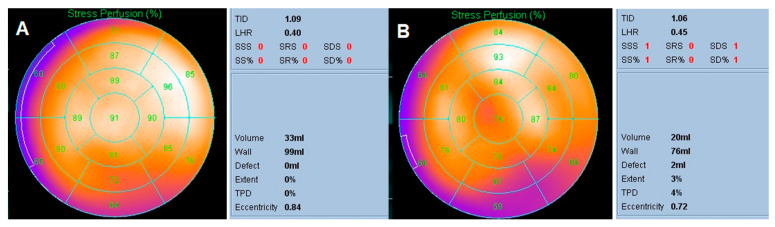
Demonstration of semi-quantitative parameters from myocardial perfusion imaging after analysis of image reconstruction. (**A**) The myocardial perfusion imaging (MPI) of a 64-year-old man who suffered from atypical chest pain. The bull’s eye revealed nearly normal perfusion on the stress phase image, with a stress total perfusion deficit (sTPD) of 0%. His cardiac catheterization one week later disclosed no significant stenosis in the 3 coronary arteries. (**B**) The MPI of a 75-year-old woman who suffered from chest pain for a period of time. The bull’s eye revealed decreased perfusion in the proximal inferior and proximal to mid-inferolateral walls of the left ventricle. The sTPD was 4%. Other parameters such as transient ischemic dilation (TID), lung-to-heart ratio (LHR), summed stress score (SSS), summed rest score (SRS) and summed difference score (SDS) were also observed. Her cardiac catheterization two weeks later disclosed a 70% stenosis in Seg 1 of the right coronary artery. SS: summed stress; SR: summed rest; SD: summed difference.

The semi-quantitative parameters were obtained by using the automated computer software AutoQUANT Version 6.5 (Cedars-Sinai, Los Angeles, CA, USA) to analyze the myocardial perfusion images. This study explored the relevant parameters of the 17-segment, five-point scoring system, TID, LHR and TPD. These parameters have high reproducibility and good standardization, which can increase the objectivity of diagnosis (Figure 1).

The perfusion territories of the three major coronary arteries of the left ventricle were divided into 17 regions on the bull’s eye projection, short axis and long axis by the method of the 17-segment, five-point scoring system. Every area had a score from 0 to 4 to evaluate the degree of perfusion defects. The higher the score, the more severe the ischemia caused by vessel stenosis (namely, 0: normal perfusion; 1: mildly decreased perfusion; 2: moderately decreased perfusion; 3: severely decreased perfusion; 4: loss of perfusion). The summed stress score (SSS) stands for the summation of the scores of all 17 segments under stress imaging. Similarly, the summed rest score (SRS) stands for the summation of the scores of all 17 segments under the resting image. The summed difference score (SDS) can be obtained by subtracting the SRS from the SSS. The SDS parameters determine whether myocardial cells are ischemic and the severity of ischemia.

When the heart is under the stress phase, the pressure in the left ventricle at the end of diastole increases and the pressure in the epicardium decreases. This causes a hypoperfusion of the subendocardial area, which results in a significant reduction in the radioactivity taken up. The image shows a thinner left ventricular myocardial wall and a larger ventricular cavity and thus reveals a picture of left ventricular dilation. TID stands for transient ischemic dilation of the left ventricle. The ratio is obtained as the stress left ventricular endocardial volume divided by the resting left ventricular endocardial volume.

The LHR is defined as the ratio of the scintigraphic counts of the left lung divided by the scintigraphic counts of the left ventricular myocardial area obtained in the stress phase image. When the coronary stenosis occurs, the increased blood flow cannot enter the left ventricle and gathers in the direction of the pulmonary vein. Radiopharmaceuticals also accumulate in the lung tissues along with the blood flow, and a “brighter” image is observed in bilateral lungs, which, in turn, leads to a higher LHR.

The TPD is a parameter that combines the severity and extent of perfusion defects. The TPD is defined as follows:

$$\mathrm{TPD}=100\%\;\times \overset{a\text{<}A}{\underset{a=0}{\sum }}\overset{p\text{<}P}{\underset{p=0}{\sum }}\mathrm{score}(a,p)/\left(4\;\times \;A\;\times \;P\right)$$

In the equation, a and p represent the radial coordinates of the polar map, while A and P represent the maximum numbers of samples in each dimension. The score (a,p) means the pixel score at the location (a,p) on the polar map. Theoretically, the maximum value for TPD was 100% for a case without visible uptake (i.e., <70% below normal) in the whole myocardium of the left ventricle. The value can be from 0 to 100%. The larger the value, the greater the severity and extent of myocardial ischemia. The TPD parameters can be subdivided into the TPD of the stress phase (stress TPD, sTPD) and resting phase (resting TPD, rTPD). The TPD of the stress phase minus that of the resting phase is the ischemic TPD parameter (ischemic TPD, iTPD).

### 2.4. Cardiac Catheterization

Cardiac catheterization (the puncture of the femoral artery of the groin, the radial artery of the wrist or the brachial artery) is to insert a special sterile catheter into the coronary artery of the heart along the direction of blood flow. It is the gold standard for diagnosing coronary artery disease and an invasive procedure in which the treatment stents can be placed. We recorded patients as positive for CAD if one or more coronary artery occlusions with diameter stenosis ≥50% in the cardiac catherization procedure were identified; otherwise, they were defined as negative for CAD.

### 2.5. Statistical Analysis

The Kolmogorov–Smirnov test was used for normality checks. Continuous variables are expressed as mean ± standard deviation. The optimal cutoff values for the variables were obtained using analysis of receiver-operating characteristics (ROC) curves with the highest Youden Index [24]. The ROC analysis for combined parameters was performed using multiple logistic regressions. The correlations between the parameters and clinical setting were analyzed using Spearman’s rank correlation test. The variable of the double-class group was tested with the Mann–Whitney U test. The statistical analysis was performed with SPSS Statistics Version 20 (International Business Machines Corporation, Armonk, NY, USA). A two-tailed *p* value < 0.05 was considered statistically significant.

## 3. Result

### 3.1. Patient Characteristics

There were 1685 patients that underwent single-day, stress and resting myocardial perfusion imaging during the period of the collection dates. A total of 130 subjects who met the inclusion criteria were enrolled in this study. Their demographic and clinical characteristics regarding age, gender and relevant clinical history are summarized in Table 1. There were 100 male (76.9%) and 30 female (23.1%) patients, with ages between 34 and 89. Regarding the clinical history, 60 (49%) subjects had type 2 diabetes, 73 (56.2%) had hypertension, 62 (47.7%) had dyslipidemia and 14 (10.8%) had end-stage renal disease. Thirty-five (26.9%) patients had a history of smoking, and 13 (10%) patients had a history of prior myocardial infarction. After cardiac catheterization examination, 83 (63.9%) patients were confirmed to have coronary artery disease.

### 3.2. Semi-Quantitative Parameters of Myocardial Perfusion Imaging

Among the 130 patients examined, the mean values of the semi-quantitative parameters related to myocardial perfusion imaging were as follows: TID, 1.06 ± 0.16; LHR, 0.38 ± 0.07; SSS, 7.49 ± 7.77; SRS, 3.86 ± 6.25; SDS, 3.18 ± 3.17; sTPD, 8.99 ± 10.98; rTPD, 7.39 ± 9.25; and iTPD, 1.60 ± 6.40.

### 3.3. Identifying the Most Discriminative Cutoff Values

**Figure 2:**
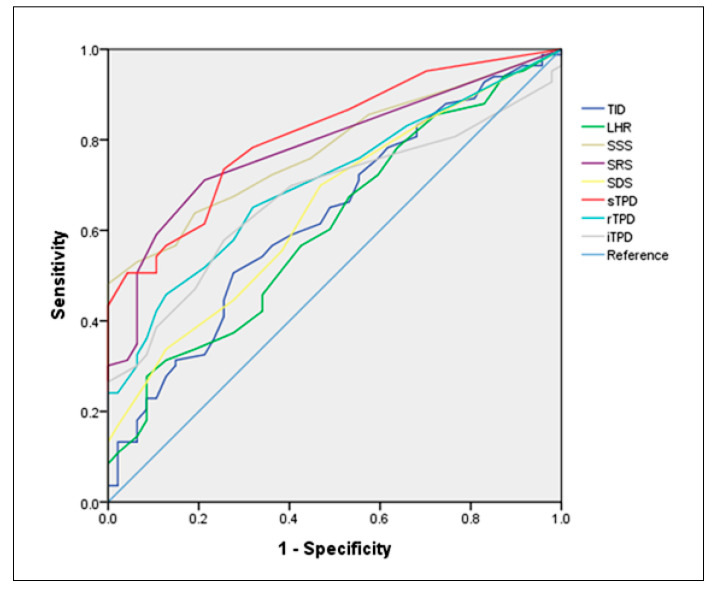
The receiver operating characteristic (ROC) curves for semi-quantitative parameters derived from myocardial perfusion imaging correlated with coronary artery diseases. rTPD: resting total perfusion deficit; iTPD: ischemic total perfusion deficit.

The correlations between the semi-quantitative parameters obtained by myocardial perfusion imaging and CAD using the ROC curve were analyzed (Figure 2). The optimal cutoff values of each semi-quantitative parameter were obtained. Statistical significance for all the parameters was noted as listed in Table 2. Among the semi-quantitative parameters, sTPD had an Area Under Curve (AUC) of 0.813 with a cutoff value of 3.5% (*p* < 0.0001; 95% CI: 0.741–0.884). The sensitivity and specificity were 73.5% and 74.5%. The SSS and SRS have equivalent AUCs (0.780, 95% CI: 0.703–0.856; 0.786, 95% CI: 0.708–0.864, respectively). Due to the overlapping 95% confidence intervals, the differences among the SSS, SRS and sTPD became insignificant. The case was the same for the pairwise comparisons of the ROC curves with sTPD. The ROC analysis for pooled parameters (combined SSS, SRS and sTPD) was further conducted and showed an AUC of 0.811 (with a 95% CI of 0.733–0.874).

### 3.4. sTPD Analysis in Different Subgroups

Using Spearman′s rank correlation test, the sTPD was positively correlated with patient age; however, the correlation was not statistically significant. Then, the Mann-Whitney U test was applied to test the difference of sTPD according to different clinical settings, as listed in Table 3. There were statistical differences in sTPD between the different gender of the subjects (*p* = 0.0010), patients who had type 2 diabetes (*p* = 0.0009), dyslipidemia (*p* = 0.0070), and end-stage renal disease (*p* = 0.029) or not.

Then we divided patients into subgroups according to gender, history of type 2 diabetes and dyslipidemia due to their statistical significance. The optimal cutoff values of sTPD in each subgroup were calculated. Further comparison of the difference of AUC, sensitivity and specificity corresponding to the optimal cutoff value of each subgroup was conducted.

All 130 subjects were classified into male or female, diabetes-positive or negative, and dyslipidemia-positive or negative subgroups. The optimal cutoff values for sTPD in each subgroup are shown in Table 4.

The results indicated that every subgroup has statistically significant results for retrieving the optimal cutoff values. The optimal cutoff values for sTPD and its corresponding sensitivity and specificity are 8.5%, 54.9% and 93.1% for all male patients; 3.0%, 66.7% and 77.8% for all female patients; 8.5%, 53.1% and 100% for diabetes-positive patients; and 8.5%, 64.4% and 94.1% for dyslipidemia-positive patients. The 95% CIs ranged from 0.588 to 0.950.

## 4. Discussion

Gated MPI with SPECT provides clinical information regarding the myocardial perfusion status, e.g., reversible or fixed perfusion defects, regional wall motion, and LV volumes/functions, as well as regional wall thickening, etc. In some clinical situations, visual interpretation may be feasible; however, there are some problems, which include the time-consuming analysis and lack of reproducibility. Thus, it depends a lot on the observer′s expertise.

Currently, a number of validated software packages, which are distributed by the main vendors of nuclear medicine imaging equipment, are available for automated quantification [25,26,27,28]. Computer-based quantitative methods have provided an important means of improving and maintaining the consistency of interpretation [29]. Based on similar principles, these software packages have provided quantitative information regarding the extent of perfusion compared against normal data files.

Based on comparison with normal limits at a regional (per vascular territory) or global (per ventricle) level, there are various quantitative parameters derived from myocardial perfusion scans. For example, the extent of a perfusion defect can be expressed as the percentage of pixels in the polar map for which the severity is greater than a predefined statistical threshold (e.g., 2 to 2.5 standard deviations below normal limits). This measurement also reflects the size of the perfusion defect. The TPD, employed by the Cedars-Sinai Quantified Perfusion SPECT (QPS) module [19], is the most commonly used single parameter to quantify the overall magnitude of hypoperfusion. It combines both pixel-based severity and extent. As well as the iTPD, the sTPD and rTPD can also be used to quantify ischemia.

This retrospective study was conducted using the correlation of semi-quantitative parameters from Tl-201 myocardial perfusion imaging and positivity for CAD. After using ROC curve analysis, sTPD, SSS and SRS had equivalent AUCs, for which the pairwise comparisons of ROC curves were insignificant. The Youden indices of these three parameters were all over 0.47. The AUC and its 95% CI for the pooled parameters (combined SSS, SRS and sTPD) are similar to those of sTPD alone. In the clinical setting, the sTPD is easily assessed, in which both pixel-based severity and extent are presented. In our report, since the case number of patients was not large, some settings of the parameters may not be statistically meaningful. Instead, the parameters were determined by the convenience of image interpretation. Thus, the sTPD was selected as the studied parameter. According to our study results, the best cutoff value for sTPD was more than 3.5%. However, the TPD calculated in the current medical software is an integer, and there will be no numbers after the decimal point. Therefore, we defined the threshold of sTPD more than 3.5% as more than 4% in the clinical setting. Under the threshold of the stress phase TPD being equal to or more than 4% for predicting CAD, the sensitivity and specificity are 73.5% and 74.5%, respectively.

There has been related research investigating different semi-quantitative parameters based on different subjects’ own conditions, and the thresholds for the diagnosis of CAD have been different. For example, among patients with type 2 diabetes, the TID value for CAD diagnosis has varied [30]. The threshold for the LVEF has also changed according to different gender and age [31]. In the current study, we focused on whether the sTPD parameters also had different thresholds based on patients with different clinical settings. Initially, using the ROC curve to analyze various variables for detecting CAD, there was statistical significance for four variables (i.e., gender, type 2 diabetes, dyslipidemia and end-stage renal disease). Not surprisingly, these variables are also regarded as risk factors for CAD [32]. Due to uneven population numbers between end-stage renal disease-positive and end-stage renal disease-negative patients, we excluded end-stage renal disease in order to avoid possible statistical bias. Therefore, the remaining three variables were divided into subgroups, and the optimal cutoff value and corresponding sensitivity and specificity for sTPD in each subgroup were calculated. In diabetes-positive and dyslipidemia-positive patients, the optimal cutoff values for sTPD were over 8.5, with AUCs of 0.828 and 0.857, respectively. The discrimination abilities were higher than those for all patients.

The results of the current study show that sTPD played a helpful role in the diagnosis of CAD. Other literature has also confirmed that sTPD was more repeatable than subjective visual interpretation [14,33]. It became one of the most important parameters for evaluating CAD in the imaging guidelines of the American Society of Nuclear Medicine (ASNC) in 2010 [34].

There are some limitations in the current study. It was a retrospective study dealing with a relatively small patient population, where male patients predominated. A pharmaceutical induced-stress protocol using dipyridamole was used rather than the treadmill induced-stress test. We used coronary angiography as the gold standard for the diagnosis of CAD, and the criterion for positivity for CAD was defined as diameter stenosis ≥50% on the coronary angiography. Gould et al. had mentioned that compensatory changes of the distal coronary vascular bed varied depended on different degrees of coronary stenosis [35]. Based on these numbers, there may be have been bias in the statistical management of some subgroups. A prospective study with larger patient populations, more subgroups based on different degrees of stenosis and other imaging modalities as references may be needed to reduce potential errors.

## 5. Conclusions

Based on the currently collected data and image acquisition conditions, the sTPD parameter has a clinical role for the diagnosis of CAD with a cutoff value of 3.5%. This result may improve the accuracy of CAD diagnosis.

## Figures and Tables

**Table 1:** Clinical characteristics of 130 patients in this study.

Variable	Value (%)
Age	
Mean ± SD	62.5 ± 12.1
Range	34–89
Gender	
Male	100 (76.9%)
Female	30 (23.1%)
Type 2 diabetes	60 (49.0%)
Hypertension	73 (56.2%)
Dyslipidemia	62 (47.7%)
End-stage renal disease	14 (10.8%)
Smoking history	35 (26.9%)
Prior myocardial infarction	13 (10.0%)
Coronary artery disease	83 (63.9%)

**Table 2:** The ideal cutoff values using ROC curve analysis for distinguishing the semi-quantitative parameters of myocardial perfusion imaging correlated with coronary artery disease.

	AUC	*p*	95% CI	Cutoff Value	Sensitivity	Specificity	95% CI
TID	0.630	0.0140	0.531–0.728	1.0	0.506	0.723	0.574–0.844
LHR	0.609	0.0400	0.509–0.708	0.4	0.277	0.915	0.796–0.976
SSS	0.780	<0.0001	0.703–0.856	8.5	0.482	1.000	0.925–1.000
SRS	0.786	<0.0001	0.708–0.864	0.5	0.711	0.787	0.643–0.893
SDS	0.650	0.0050	0.554–0.745	1.5	0.699	0.532	0.381–0.679
sTPD	0.813	<0.0001	0.741–0.884	3.5	0.735	0.745	0.597–0.861
rTPD	0.705	<0.0001	0.617–0.793	3.5	0.651	0.681	0.529–0.809
iTPD	0.675	0.0010	0.584–0.766	0.5	0.578	0.745	0.597–0.861

**Table 3:** Differences in sTPD according to different clinical settings analyzed using Mann–Whitney U tests.

	sTPD	
	Median (Interquartile Range)	*p* Value
Gender		0.0014 *
Male	5.0 (15.5)	
Female	2.0 (4.0)	
Type 2 diabetes		0.0010 *
Yes	6.5 (18.5)	
No	2.0 (8.0)	
Hypertension		0.1522
Yes	4.0 (9.0)	
No	5.0 (16.0)	
Dyslipidemia		0.0078 *
Yes	8.0 (19.0)	
No	3.0 (6.0)	
End-stage renal disease		0.0302 *
Yes	8.5 (31.0)	
No	4.0 (9.5)	
History of smoking		0.8351
Yes	4.0 (15.0)	
No	4.0 (9.0)	
Prior myocardial infarction		0.1854
Yes	10.0 (20.3)	
No	4.0 (10.3)	

* statistically significant.

**Table 4:** The ideal cutoff values for sTPD from myocardial perfusion imaging correlated with coronary artery disease in different subgroups of patients using ROC curve analysis.

	AUC	*p* Value	95% CI	Optimal Cutoff Value	Sensitivity	Specificity	95% CI
All	0.813	<0.0001	0.741–0.884	3.5	0.735	0.745	0.597–0.861
Male	0.811	<0.0001	0.727–0.895	8.5	0.549	0.931	0.772–0.992
Female	0.766	0.0150	0.588–0.944	3.0	0.667	0.778	0.524–0.936
Type 2 diabetes							
Positive	0.828	<0.0001	0.715–0.942	8.5	0.531	1.000	0.715–1.000
Negative	0.777	<0.0001	0.667–0.886	2.5	0.706	0.722	0.548–0.858
Dyslipidemia							
Positive	0.857	<0.0001	0.764–0.950	8.5	0.644	0.941	0.713–0.999
Negative	0.767	<0.0001	0.656–0.878	3.5	0.658	0.767	0.577–0.901
